# Supplementary material for: Balanced steady state free precession enables high-resolution dynamic 3D Deuterium Metabolic Imaging of the human brain at 7T
Source: medRxiv. 2025 Feb 7:2025.02.06.25321580. Preprint. [Version 1] doi: 10.1101/2025.02.06.25321580 (PMC11838661; doi:10.1101/2025.02.06.25321580)
Supplement: Supplement 1 [file NIHPP2025.02.06.25321580v1-supplement-1.pdf]

medRxiv preprint doi: https://doi.org/10.1101/2025.02.06.25321580; this version posted February 7, 2025. The copyright holder for this preprint (which was not certified by peer review) is the author/funder, who has granted medRxiv a license to display the preprint in perpetuity.
It is made available under a CC-BY 4.0 International license .

Supplemental Digital Content

medRxiv preprint doi: https://doi.org/10.1101/2025.02.06.25321580; this version posted February 7, 2025. The copyright holder for this preprint (which was not certified by peer review) is the author/funder, who has granted medRxiv a license to display the preprint in perpetuity.
It is made available under a CC-BY 4.0 International license .

Minimum Reporting Standards in MR Spectroscopy (according to Lin et al. NMR Biomed 2021)	
**1. Hardware**	
a. Field strength [T]	7T
b. Manufacturer	Siemens
c. Model (software version if available)	Magnetom dot Plus
d. RF coils: nuclei (transmit/ receive), number of channels, type, body part	$^{2}H/^{1}H$ dual tuned quadrature birdcage head coil, transmit/receive, 1 channel, (Stark Contrast MRI Coils Research, Germany)
e. Additional hardware	N/A
**2. Acquisition**	
a. Pulse sequence	3D FID-acquire density-weighted concentric ring trajectory (CRT) MRSI & 3D multi-echo balanced Steady State Free Precession (bSSFP) density-weighted concentric ring trajectory (CRT) MRI
b. Volume of Interest (VOI) locations	whole-brain, unlocalized excitation using rectangular RF pulse with 86° (FID-CRT) & ±50° (bSSFP-CRT) flip angle
c. Nominal VOI size [cm^3, mm^3]	FOV 200x200x192 mm^3
d. Repetition Time (TR), Echo Time (TE) [ms, s]	FID-CRT: TR=290 ms / 2 ms acquisition delay<br>bSSFP-CRT: TR = 23 ms / 2 ms acquisition delay
e. Total number of Excitations or acquisitions per spectrum	FID-CRT: 43 circles (7 min acquisition time)<br>bSSFP-CRT: 107 circles, 2 averages (matched 7 min acquisition time)
In time series for kinetic studies	N/A
i. Number of Averaged spectra (NA) per time-point	N/A
ii. Averaging method (e.g. block-wise or moving average)	N/A
iii. Total number of spectra (acquired / in time-series)	N/A
f. Additional sequence parameters (spectral width in Hz, number of spectral points, frequency offsets); If STEAM: Mixing Time TM; If MRSI: 2D or 3D, FOV in all directions, matrix size, acceleration factors	FID-CRT: BW: 380 Hz, 96 spectral points, 22x22x21<br>bSSFP-CRT: BW: 285 Hz, 5 echoes, 22x22x21 & 28x28x27
g. Water Suppression Method	No water suppression
h. Shimming Method, reference peak, and thresholds for "acceptance of shim" chosen	Standard shim + manual adjustment, ^{1}H water peak < 40 Hz, ^{2}H water peak < 30 Hz Region: whole brain
i. Triggering or motion correction method (respiratory, peripheral, cardiac triggering, incl. device used and delays)	-
**3. Data analysis methods and outputs**	
a. Analysis software	FID-CRT: LCModel 6.3-1<br>bSSFP-CRT: inhouse developed k-space IDEAL algorithm in MATLAB 2017
b. Processing steps deviating from quoted reference or product	N/A
c. Output measure (e.g. absolute concentration, institutional units, ratio)	concentration estimation in mM
d. Quantification references and assumptions, fitting model assumptions	Simulated in NMRScope-B
**4. Data Quality**	
a. Reported variables (SNR, Linewidth (with reference peaks))	SNR was calculated using voxel-wise mean and standard deviation across 5 repeated measurements
b. Data exclusion criteria	CRLBs > 50 % for water, Glc, Glx, no CRLB threshold for first time point
c. Quality measures of postprocessing Model fitting (e.g. CRLB, goodness of fit, SD of residual)	CRLB for FID-CRT datasets<br>None for bSSFP-CRT datasets
d. Sample Spectrum	-

Supplemental Digital Content Table 1: Minimum Reporting Standards for in vivo MR Spectroscopy

Note. – Parameters 7T DMI**,** CRLB = Cramér-Rao lower bounds; FID = free induction decay; CRT =

medRxiv preprint doi: https://doi.org/10.1101/2025.02.06.25321580; this version posted February 7, 2025. The copyright holder for this preprint (which was not certified by peer review) is the author/funder, who has granted medRxiv a license to display the preprint in perpetuity.
It is made available under a CC-BY 4.0 International license .

concentric ring trajectory; FOV = field of view; FWHM = full-width-at-half-maximum; Glx = Glutamate+Glutamine; Glc = Glucose; SNR = signal-to-noise ratio; VOI = volume of interest.

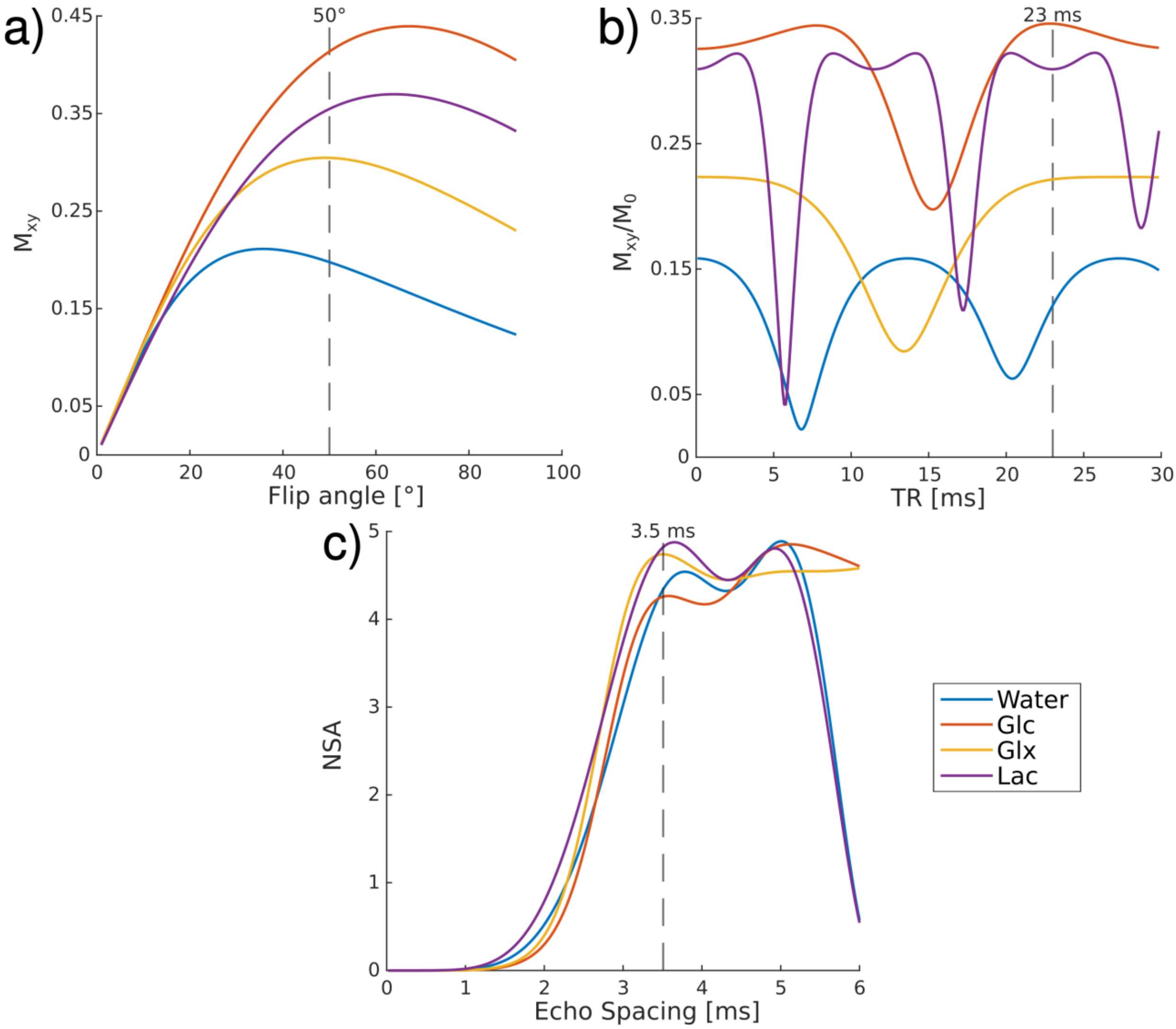

Supplemental Digital Content Figure 1: Parameter optimization according to D. Peters et al., 2021, and pulse design of the balanced Steady State Free Precession sequence combined with concentric ring trajectory readout.

a) Calculation of optimal flip angle for the metabolites of interest, ^{2}H-Glucose (Glc), ^{2}H-Glutamate+Glutamine (Glx), ^{2}H-Lactate (Lac) and ^{2}H-Water. As a compromise between signal gain of all metabolites and limitation of Specific Absorption Rate (SAR), a flip angle of 50° was chosen.

medRxiv preprint doi: https://doi.org/10.1101/2025.02.06.25321580; this version posted February 7, 2025. The copyright holder for this preprint (which was not certified by peer review) is the author/funder, who has granted medRxiv a license to display the preprint in perpetuity.
It is made available under a CC-BY 4.0 International license .

b) Repetition time (T_R), calculated with A2, was set to 23ms to avoid banding artifacts for all metabolites, and accommodate 5 echoes for metabolite separation using an IDEAL algorithm.

c) Echo spacing (ΔT_E) was set to 3.5ms to optimize phase difference between the four metabolites of interest for optimal metabolite separation using an IDEAL algorithm, expressed with the number of signal averages(NSA), while keeping T_R as short as possible for 5 echoes.

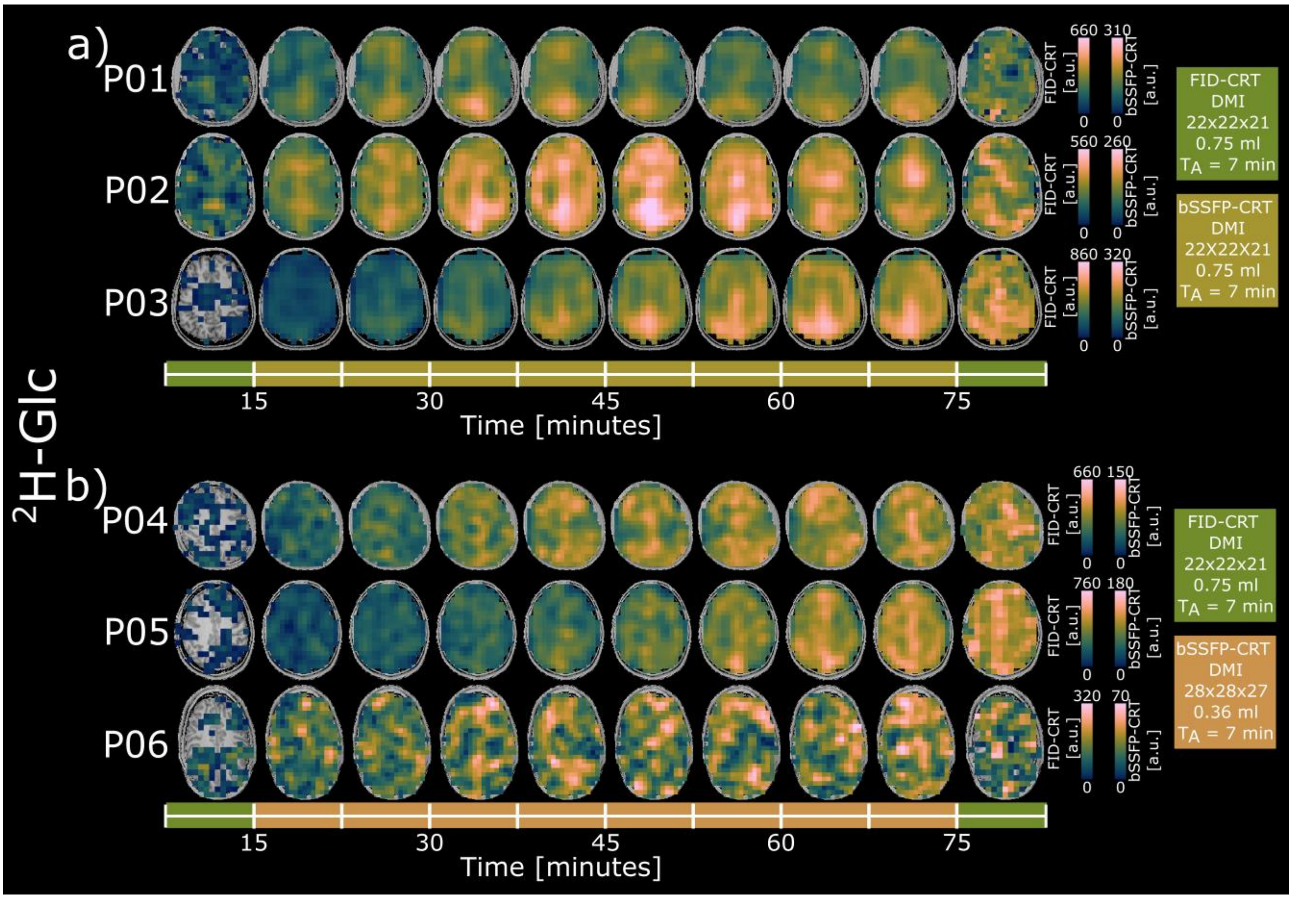

Supplemental Digital Content Figure 2: Time courses of axial ^{2}H-Glucose (Glc) maps given in arbitrary units (a.u.) from all participants (a: both acquisitions 0.75ml isotropic resolution, b: FID-CRT 0.75ml, bSSFP-CRT 0.36ml isotropic resolution), detected using deuterium metabolic imaging (DMI) with the Free Induction Decay Concentric Ring Trajectory (FID-CRT, green) and the balanced Steady State Free Precession Concentric Ring Trajectory (bSSFP-CRT, olive green and orange) sequences at 7T. Missing voxels in the metabolic maps do not contain a value (NaN: not a number).

medRxiv preprint doi: https://doi.org/10.1101/2025.02.06.25321580; this version posted February 7, 2025. The copyright holder for this preprint (which was not certified by peer review) is the author/funder, who has granted medRxiv a license to display the preprint in perpetuity.
It is made available under a CC-BY 4.0 International license .

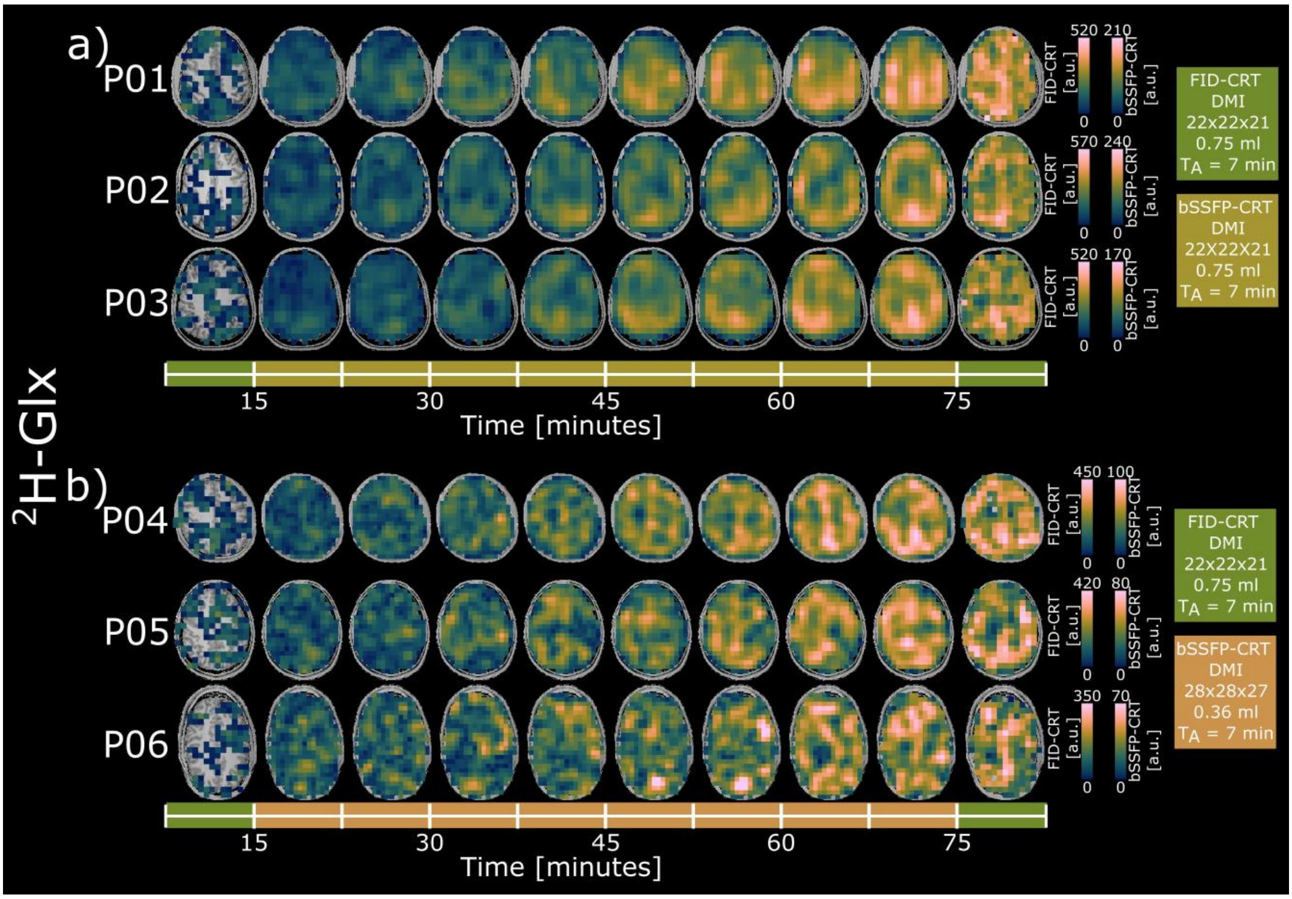

Supplemental Digital Content Figure 3: Time courses of axial ^{2}H-labeld Glutamate+Glutamine (Glx) maps given in arbitrary units (a.u.) from all participants (a: both acuqisitions 0.75ml isotropic resolution, b: FID-CRT 0.75ml, bSSFP-CRT 0.36ml isotropic resolution), detected using deuterium metabolic imaging (DMI) with the Free Induction Decay Concentric Ring Trajectory (FID-CRT, green) and the balanced Steady State Free Precession Concentric Ring Trajectory (bSSFP-CRT, olive green and orange) sequences at 7T. Missing voxels in the metabolic maps do not contain a value (NaN: not a number).

medRxiv preprint doi: https://doi.org/10.1101/2025.02.06.25321580; this version posted February 7, 2025. The copyright holder for this preprint (which was not certified by peer review) is the author/funder, who has granted medRxiv a license to display the preprint in perpetuity.
It is made available under a CC-BY 4.0 International license .

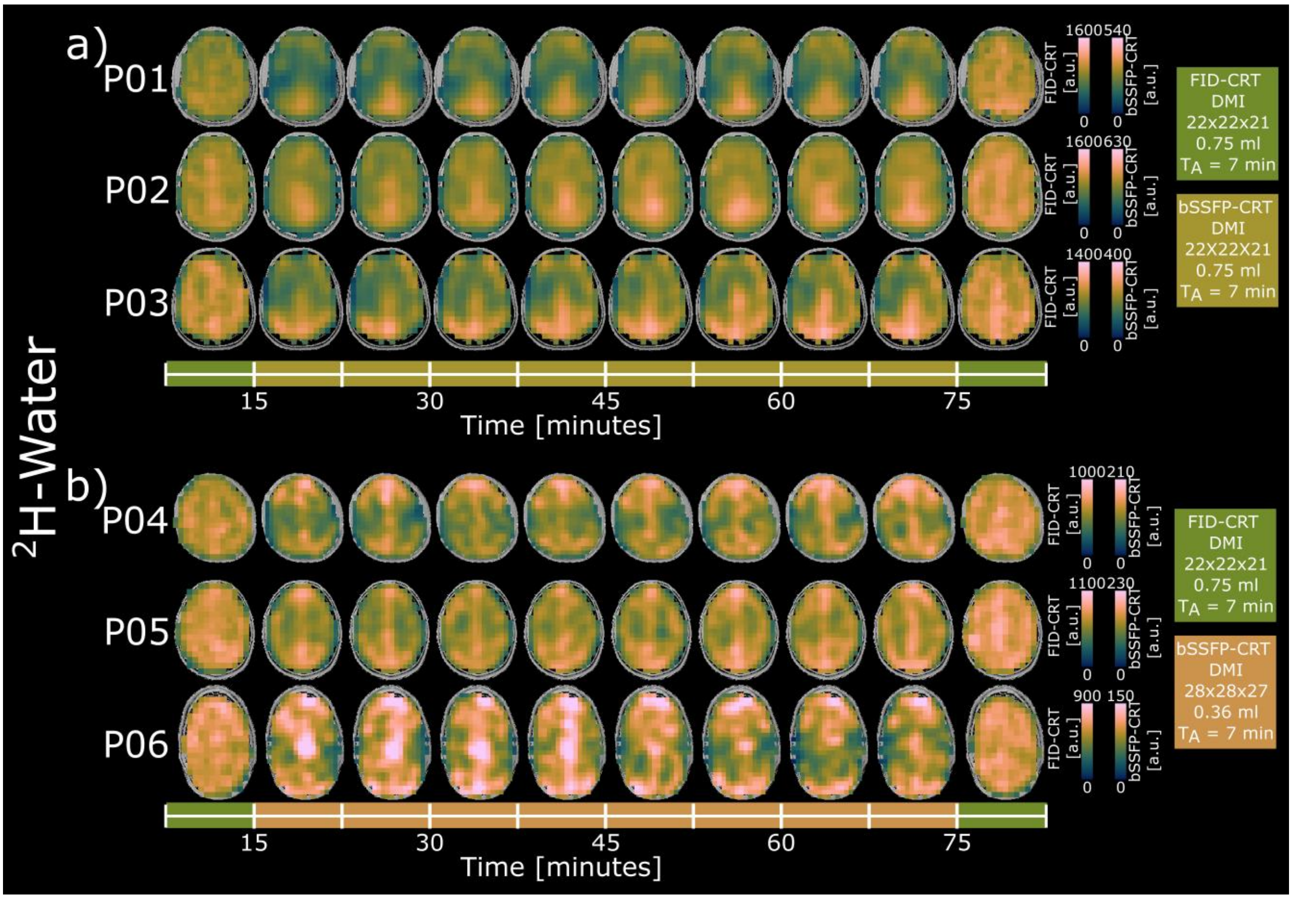

Supplemental Digital Content Figure 4: Time courses of axial ^{2}H-water maps given in arbitrary units (a.u.) from all participants (a: both acquisitions 0.75ml isotropic resolution, b: FID-CRT 0.75ml, bSSFP-CRT 0.36ml isotropic resolution), detected using deuterium metabolic imaging (DMI) with the Free Induction Decay Concentric Ring Trajectory (FID-CRT, green) and the balanced Steady State Free Precession Concentric Ring Trajectory (bSSFP-CRT, brown and orange) sequences at 7T.

medRxiv preprint doi: https://doi.org/10.1101/2025.02.06.25321580; this version posted February 7, 2025. The copyright holder for this preprint (which was not certified by peer review) is the author/funder, who has granted medRxiv a license to display the preprint in perpetuity.
It is made available under a CC-BY 4.0 International license .

Appendix 1: Absolute concentration estimation in mM units of ^{2}H resonances detected using Deuterium Metabolic Imaging (DMI) at 7T with Free Induction Decay Concentration Ring Trajectory (FID-CRT) and balanced Steady State Free Precession Concentric Ring Trajectory (bSSFP-CRT). The amplitude of the deuterated metabolites was referenced voxel wise to the amplitude of the deuterated water signals. The amplitude ratios were corrected for the relaxation factors (equations A1 and A2) and fractional water content for gray and white matter (GM, WM) and cerebrospinal fluid (CSF) and tissue water content with d_{GM}=0.78, d_{WM}=0.65 and d_{CSF}=0.97. Fractional water content of CSF was excluded for ^{2}H-Glx concentration estimation. Label loss of ^{2}H-Glx was corrected according to de Graaf et al., ACS Chemical Neuroscience, 2021 (assuming on average approximately 40%).

$$R_{\mathrm{M,FID-CRT}} = \sin\alpha * e^{-T_{\mathrm{E}}/T_2} * \left(1 - e^{-T_{\mathrm{R}}/T_1}\right) \qquad \text{(A1)}$$

$$R_{\mathrm{M,bSSFP-CRT}} = \sin\alpha * e^{-T_{\mathrm{R}}/2\cdot T_2} * \left(1 - e^{-T_{\mathrm{R}}/T_1}\right) * \frac{1}{1 - \left(e^{-T_{\mathrm{R}}/T_1} - e^{-T_{\mathrm{R}}/T_2}\right) * \cos\alpha - e^{-T_{\mathrm{R}}/T_1} * e^{-T_{\mathrm{R}}/T_2}} \qquad \text{(A2)}$$
